# An integrated transcriptomic analysis of brain aging and strategies for healthy aging

**DOI:** 10.3389/fnagi.2024.1450337

**Published:** 2024-12-05

**Authors:** Haiying Liu, Xin Nie, Fengwei Wang, Dandan Chen, Zhuo Zeng, Peng Shu, Junjiu Huang

**Affiliations:** ^1^MOE Key Laboratory of Gene Function and Regulation, State Key Laboratory of Biocontrol, Guangdong Key Laboratory of Pharmaceutical Functional Genes, School of Life Sciences, Sun Yat-sen University, Guangzhou, Guangdong, China; ^2^HBN Research Institute and Biological Laboratory, Shenzhen Hujia Technology Co., Ltd., Shenzhen, Guangdong, China; ^3^State Key Laboratory Basis of Xinjiang Indigenous Medicinal Plants Resource Utilization, Xinjiang Technical Institute of Physics and Chemistry, Chinese Academy of Sciences, Ürümqi, Xinjiang, China

**Keywords:** aging gene, brain aging, neurodegenerative diseases, retard aging, transcriptome

## Abstract

**Background:**

It is been noted that the expression levels of numerous genes undergo changes as individuals age, and aging stands as a primary factor contributing to age-related diseases. Nevertheless, it remains uncertain whether there are common aging genes across organs or tissues, and whether these aging genes play a pivotal role in the development of age-related diseases.

**Methods:**

In this study, we screened for aging genes using RNAseq data of 32 human tissues from GTEx. RNAseq datasets from GEO were used to study whether aging genes drives age-related diseases, or whether anti-aging solutions could reverse aging gene expression.

**Results:**

Aging transcriptome alterations showed that brain aging differ significantly from the rest of the body, furthermore, brain tissues were divided into four group according to their aging transcriptome alterations. Numerous genes were downregulated during brain aging, with functions enriched in synaptic function, ubiquitination, mitochondrial translation and autophagy. Transcriptome analysis of age-related diseases and retarding aging solutions showed that downregulated aging genes in the hippocampus further downregulation in Alzheimer’s disease but were effectively reversed by high physical activity. Furthermore, the neuron loss observed during aging was reversed by high physical activity.

**Conclusion:**

The downregulation of many genes is a major contributor to brain aging and neurodegeneration. High levels of physical activity have been shown to effectively reactivate these genes, making it a promising strategy to slow brain aging.

## 1 Introduction

Aging is a complex multifactorial process involving changes of DNA modification, gene transcription, protein translation and somatic mutation, leading to tissue and cell deterioration. As the development of large-scale high-throughput technologies, it allows researchers to explore the enigmatic process of aging comprehensively by “omics” approaches, especially the transcriptomic, beneficial from the next generation sequencing and microarray. According to previous studies, large amount of genes changed during aging in different tissues (Peters et al., 2015; Fleischer et al., 2018). In a meta-analysis of whole-blood gene expression profiles including 14,983 individuals, 1497 genes were found to associate with age (Peters et al., 2015). In addition, an ensemble machine learning method was developed to predict age using transcriptomes of human dermal fibroblasts (Fleischer et al., 2018), suggesting a good correlation between a set of genes and age.

Aging leads to deterioration of tissues and cells, and is the primary risk factor for age-related diseases, such as neurodegenerative diseases, cardiovascular diseases, etc. How does aging drive the age-related diseases occurrence is an important question for anti-aging and age-related diseases prevention. According to previous reports, deregulation of aging genes is probably one of the reasons for age-related diseases development. For example, the famous longevity gene family SIRT was reported to decrease during aging and associate with many age-related diseases (Hsu et al., 2010; Lee et al., 2018). Cardiac specific overexpression of SIRT1 in mice showed delayed age-dependent cardiomyopathies (Hsu et al., 2010), and SIRT3 overexpression ameliorated neurodegeneration in AD (Lee et al., 2018). These studies provide a clue that gradual deregulation of aging genes provided convenience for age-related diseases progress, and artificially reverse the deregulation of the genes alleviate age-related diseases. Furthermore, it has been reported that caloric restriction could prevent age-related hearing loss by activation of SIRT3 (Someya et al., 2010), implying that anti-aging activities probably retard aging by reverse the deregulation of key aging genes. Yet, limited number of aging genes has been proved impact diseases occurrence in the elderly.

In this study, we aim to screen for brain aging genes and explore the contribution of aging genes to neurodegeneration using transcriptome profiles from Genotype-Tissue Expression (GTEx) project and Gene Expression Omnibus (GEO) database. We also screened for the aging genes that were reversed by anti-aging actions such as physical activities in the hippocampus.

## 2 Materials and methods

### 2.1 Aging gene screen using transcriptome from GTEx

GTEx transcriptome files and donor information were download from GTEx website.^1^ Data from donor aged 40–60 s were included for aging gene screening. The correlation between each protein coding genes (18853 genes) and age were estimated respectively by Pearson correlation in 32 tissues, including 13 tissues from brain. The resulted *r*-values were clustered between 32 tissues using the R package “pheatmap.”

Considering that a large number of genes decrease in the brain during aging, we used a more stringent criterion to screen genes negatively associated with age during brain aging. To be more specific, the genes with *r* > 0 and *p* < 0.05 were defined as “upregulated aging genes,” whereas the genes with *r* < 0 and *p* < 0.01 were defined as “downregulated aging genes.”

### 2.2 Aging gene validation using datasets from GEO

Two sets of front cortex transcriptome profiles (GSE30272, GSE53890) were downloaded from GEO database to validate the aging genes screened from the GTEx project. The data were analyzed with Microarray Suite version 5.0, and normalized by global scaling. The normalized expression data were log-transformed. Data from subjects older than 20 years were used for Pearson correlation analysis between ages and normalized gene expression levels. To match the data form, the age was also log-transformed before correlation analysis.

### 2.3 Neurodegeneration gene screen using datasets from GEO

Three transcriptome datasets of brain tissues (GSE48350, GSE20168, GSE20292) (Berchtold et al., 2008; Zheng et al., 2010) from Alzheimer’s or Parkinson’s disease (AD or PD) patients were used for explore whether common aging genes in brain were also deregulated in neurodegenerative diseases. Data from individual with matched age and gender were chosen for comparison between patients and control subjects in hippocampus, front cortex or substantia nigra respectively. Sample information was described in Supplementary Table 1. All of these datasets were generated by microarray, and normalized by RMA. To obtain comparable data within each brain area, the individual transcript data were further normalized within each respective brain area to the mean intensity measurements of the control subject samples for that brain area and transcript. Then the fold changes between AD or PD patients and control subjects were calculated using R package “edgeR” (Robinson et al., 2010).

### 2.4 Reversed genes screen by anti-aging action using datasets from GEO

Three transcriptome datasets from anti-aging action subjects and control (GSE110298, GSE95624, GSE107894) (Bolton et al., 2017; Kulkarni et al., 2018; Berchtold et al., 2019) were used for explore whether tissue specific aging genes were reversed by anti-aging actions, including physical activity, calorie restriction and metformin treatment. In dataset GSE110298, the subjects were divided into three groups of high, moderate or low level of physical activity according to previous report (Berchtold et al., 2019). In dataset GSE95624, the calorie restriction subjects were divided into two groups of good or bad controller according to their different maintenance of weight loss during calorie restriction (Bolton et al., 2017). These information are important because they were key factors that influence anti-aging outcomes. The age and gender were matched in each dataset, and other information was list in Supplementary Table 1. Datasets generated by microarray were analyzed by R package “edgeR,” whereas RNAseq datasets were analyzed by R package “DESeq” (Anders and Huber, 2010), both with default normalizations.

### 2.5 Digital cell classification by CIBERSORTx

CIBERSORTx is a digital cytometry method that enables estimation of cell type abundances from bulk tissue transcriptomes (Newman et al., 2019). Briefly, this method generates signature matrix using single-cell RNA sequencing (scRNAseq) data and then dissects cellular content from bulk sequence of mixture samples. In this study, we generated signature matrix using scRNAseq data GSE84465, which contains the major central nervous system (CNS) cell types [neurons, oligodendrocytes, astrocytes, microglia (immune cells), vascular cells, oligodendrocyte progenitor cells (OPCs)] (Darmanis et al., 2017). Then we dissect the cellular content of hippocampus from bulk sequence data of GTEx and GSE110298. The parameters used for this analysis were B-mode batch correction and relative fraction mode for cell type dissection.

To get cell type-specific gene expression profiles in GTEx and GSE110298, high-resolution expression imputation in CIBERSORTx were performed with 3413 negative aging genes in hippocampus.

### 2.6 Cell culture

SH-SY5Y and A549 were obtained from American Type Culture Collection (Manassas, VA). A549 cells were cultured using DEME (GIBCO) high glucose medium, whereas SH-SY5Y cultured in DMEM/F12, both containing 10% fetal bovine serum (GIBCO) and 100 U/ml penicillin/streptomycin (GIBCO). Cells were cultured at 37°C and 5% CO2.

### 2.7 SA-β-gal staining

SH-SY5Y and A549 cells were seeded with 0.03 million cells per well in a six-well plate and treated with Bleomycin (25 μg/ml, Selleck) for 72 h to induce senescence. Cellular senescence was detected by SA-β-gal staining using a commercial kit (Beyotime, China) according to the manufacturer’s instructions.

For cells that GPCPD1 or TMEM169 were knocked down, SiRNAs were transfected into A549 cells 6 h before bleomycin treatment using Lipofectamine RNAiMAX reagent (Thermo Fisher Scientific) according to the manufacturer’s instruction. siRNA sequences: GPCPD1-1: 5′- GCCCGUGGUAUAUCAUGAU-3′; GPCPD1-2: 5′- GCCUGAACAACCAAAUAUA-3′; TMEM169-1: 5′- GUUGCUUGAAUCCGACAAU-3′; TMEM169-2: 5′- GUGGAGAUCAGCCUAAAGA-3′.

### 2.8 RNA extraction and real-time quantitative RT-PCR

Total RNA was extracted using Trizol extraction method. RNA was reverse transcribed using reverse transcription kit (AU311, TransGen Biotech), and qPCR was performed with a LightCycler 480 Real-Time PCR system (Roche) using the RealStar Power SYBR Mixture (GenStar). Data were analyzed using the comparative Ct (2-ΔΔCt) method, with *GAPDH* as internal reference. The following primers were used for amplification: *GAPDH*-forward: 5′- AGCCACATCGCTCAGACAC -3′; *GAPDH* -reverse: 5′- GCCCAATACGACCAAATCC -3′; *GPCPD1*-forward: 5′- GTTTT TGCGATATGTGGAAGCTG -3′; *GPCPD1* -reverse: 5′- AGCGA TACTGAACTGATACTCCT -3′; *TEME169*-forward: 5′- ATCAGC CTAAAGAGGAGGAGG -3′; *TMEM169*-reverse: 5′- GGTCCCA GTTAAGGTGACGTAG -3′; *ACTB*-forward: 5′- CATGTACGTT GCTATCCAGGC-3′; *ACTB*- reverse: 5′- CTCCTTAATGTCAC GCACGAT-3′.

### 2.9 Quantification and statistical analysis

All experiments in this study have been performed with at least three independent biological replicates. GraphPad Prism 8 was used for statistical analysis. Results are shown as mean ± SEM and the unpaired Student’s two-tailed *t*-test was used to determine the statistical significance (*P < 0.05; **P < 0.01; ***P < 0.001). For every Figure, statistical tests are justified as appropriate.

## 3 Results

### 3.1 Brain aging transcriptomes are distinct from body aging transcriptomes

**FIGURE 1:**
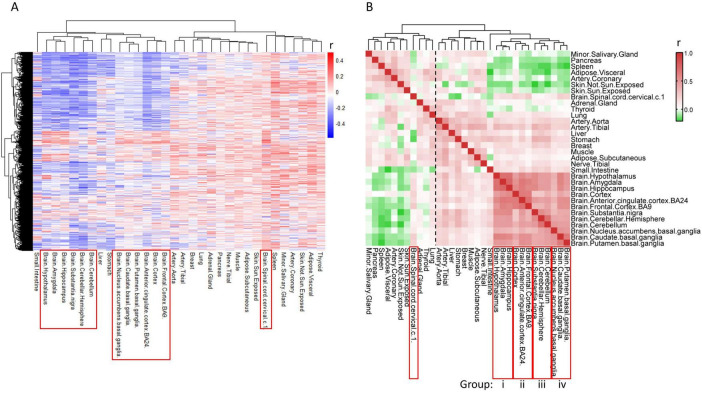
Alteration of gene expression levels is different between brain and body tissues during aging. **(A)** Heat map of *r*-values of each gene to age. Gene expression profiles of tissues were downloaded from GTEX, and the association of gene levels to age were calculated by Pearson correlation analysis. Each row represents one gene, while each column represents one tissue. **(B)** Heat map of *r*-values between tissues. Aging transcriptomes of each tissue (each column of panel **A**) was compared to the other tissues using Pearson correlation analysis.

Throughout the aging process, it is expected that there exists a set of genes defined as “aging genes”, which experience alterations in their expression levels as individuals age. To identify common patterns in transcriptomes during aging and pinpoint these aging-related genes, we utilized RNA sequence data from the Genotype-Tissue Expression (GTEx) project, which encompasses transcriptomic data across various tissues from hundreds of individuals (Melé et al., 2015). We selected 32 tissues from individuals aged 40–69 for subsequent analysis, comprising 13 brain tissues and 19 body tissues (Supplementary Table 2). Pearson correlation analysis was performed to assess the relationship between 18,853 protein-coding genes and age using 4,776 samples. The heatmap of correlation coefficients (*r*-values) revealed significant differences in gene expression alterations between brain tissues and body tissues during the aging process (Figure 1A). More specifically, the expression levels of numerous genes decreased during aging (as indicated by the blue region with *r* < 0) in the brain tissues (excluding the Spinal cord), whereas only a limited number of genes exhibited decreased expression in body tissues (Figure 1A).

We further assessed the similarity of gene expression alterations among the 32 tissues during aging using Pearson correlation analysis. The correlation coefficients between brain and body tissues exhibit diversity, with body tissues primarily clustering into two groups (Figure 1B, dashed line), displaying negative or nearly no correlation with brain tissues. In contrast, brain tissues exhibit positive correlations with each other and form a cohesive cluster, suggesting that most genes undergo similar changes during aging in brain tissues, except for the Spinal cord (Figure 1B).

Upon closer examination of the sub-clusters within brain tissues, we observed that the twelve brain tissues in the main cluster could be further subdivided into four groups, each comprising three brain tissues (Figure 1B, i–iv). Gene alterations during aging are more consistent among tissues within the same group. This grouping scheme was subsequently utilized for screening aging-related genes. The Spinal cord stands out as distinct from the other 12 brain tissues and is therefore excluded from further analysis.

### 3.2 Genes and functions deregulated in brain aging

Before screening for aging genes in each group, we initially validated the brain aging genes using datasets from GEO. There are limited datasets of human brain, with most from front cortex. Two front cortex transcriptome datasets (GSE30272 and GSE53890 in Supplementary Table 1; Colantuoni et al., 2011; Lu et al., 2014) were selected due to their large sample sizes and matched age ranges. The Pearson correlation between gene expression and age was calculated using data form healthy individuals older than 20 years. The results showed that 61 of the 85 aging genes that positively correlated with age in at least three brain tissues were also positively correlated with age in at least one of the validation datasets (Supplementary Table 3). Similarly, 369 of the 477 aging genes that negatively correlated with age in at least six brain tissues were negatively associated with age in at least one of the validation datasets (Supplementary Table 4). These findings suggest that the brain aging genes identified in this study are reliable.

**FIGURE 2:**
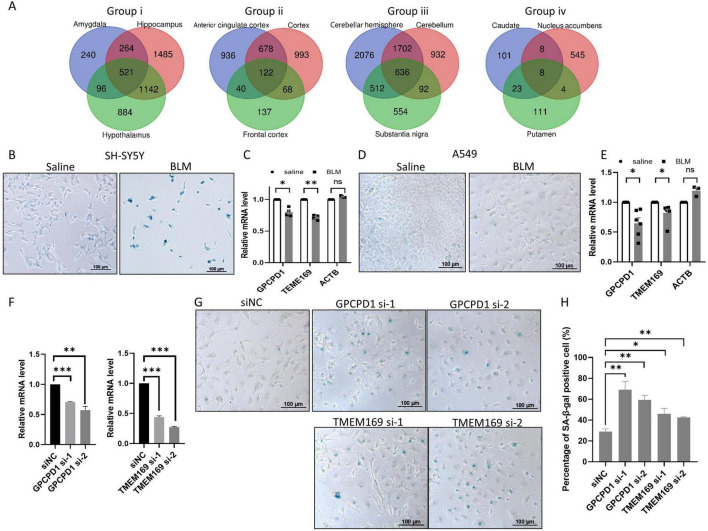
Knockdown of downregulated aging genes accelerates stress-induced senescence. **(A)** The number of group down aging genes in each group. Venn diagrams of negative aging genes in group i to iv, respectively, with the number of group down aging genes in the center. **(B,C)** The expression levels of GPCPD1 and TMEM169 are downregulated in stress induced senescent cell. Neuroblastoma cell SH-SY5Y were treated with 25 μg/mL bleomycin (BLM) for 72 h, and senescence was detected by SA-β-gal staining **(B)**, GPCPD1 and TMEM169 were detected by qRT-PCR **(C)**. **(D,E)** The experiments of panels **(B,C)** have been repeated in A549 cell. **(F–H)** GPCPD1 and TMEM169 depletion promotes stress-induced senescence. GPCPD1 and TMEM169 were depleted by siRNAs in A549 cells **(F)**, and cells were treated with BLM for 72 h. SA-β-gal staining was performed **(G)**, and the positive cell rates were counted **(H)**. Student’s *t*-test, *p < 0.05; **p < 0.01; ***p < 0.001.

Then we screened for overlapping aging genes within each group as shown in Figure 1B, and defined these genes as “group up/down aging genes.” Since most of the genes are negatively correlated with age in the brain as shown in Figure 1A, thousands of genes are decreased during aging with rigorous discipline (*p* < 0.01) except tissues in group iv and front cortex, whereas only dozens or hundreds of genes are increased with mild discipline (*p* < 0.05) (Supplementary Table 5). By screening overlapping downregulated aging genes within groups, many group down aging genes are found, with 521, 122, 636 and 8 in group i, ii, iii and iv respectively (Figure 2A). Among the group down aging genes, some genes were downregulated in all or three groups (Supplementary Table 6). Cellular senescence is a hallmark of individual aging, with accumulated senescent cells in multiple tissues of old people (Di Micco and Krizhanovsky, 2021), so does in brain aging(Melo Dos Santos et al., 2024). It is possible that these aging genes regulate cellular senescence during aging. Therefore, we chose top two of previously unreported potential aging genes (*GPCPD1* and *TMEM169*) to test whether they deregulated in neuroblastoma cell line SH-SY5Y and lung cancer cell line A549 during cell senescence. After inducing cell senescence through BLM treatment, we discovered that the expression levels of *GPCPD1* and *TMEM169* are notably downregulated in both cell lines whereas housekeeping gene *ACTB* did not changed (Figures 2B–E). Subsequently, we employed siRNAs to knock down these two genes. The depletion of *GPCPD1* and *TMEM169* resulted in the promotion of BLM-induced cell senescence (Figures 2F–H). These results suggest that the common aging genes identified in this study play crucial roles in regulating the aging process.

**FIGURE 3:**
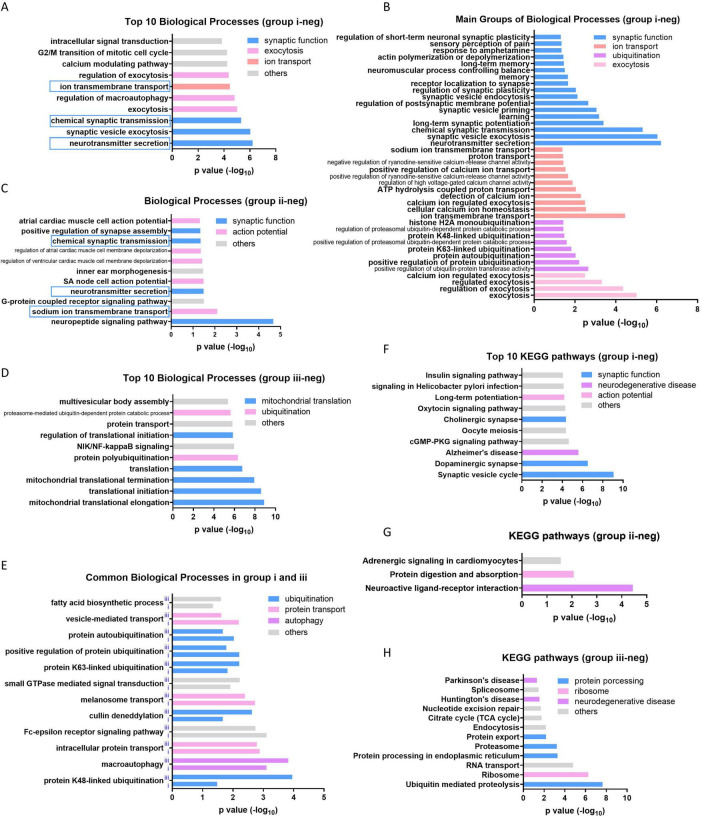
Enriched function of group down aging genes in three groups. **(A)** Top 10 biological processes enriched by group i down aging genes. **(B)** The most enriched four categories of biological process in group i. **(C)** All of the biological processes enriched by group ii down aging genes. **(D)** Top 10 biological processes enriched by group iii down aging genes. **(E)** The overlapped biological processes enriched in both group i and iii. **(F–H)** Top 10 or all of the KEGG pathways enriched in group i, ii, and iii, respectively.

The Gene Ontology analysis of 521 downregualted genes in group i showed that the functions related to synaptic transmission and neuron signaling transduction are prominently enriched, including “synaptic function,” “exocytosis” and “ion transport” (Figures 3A, B). The neuron related functions are also enriched by 122 downregualted genes in group ii (Figure 3C), whereas the mitochondrial translation associated terms are enriched as top biological processes in group iii (Figure 3D). Interestingly, multiple biological processes related to “ubiquitination,” “protein transport,” “autophagy” and others are enriched in both group i and group iii (Figure 3E), indicating widespread dysregulation of these functions across various brain tissues during the aging process. It consistent with the previous reports that the disorder of the above processes is the leading cause of neuron degeneration during aging (Bingol and Sheng, 2011; Area-Gomez et al., 2019; Stavoe and Holzbaur, 2019). It is also supported by the KEGG pathway analysis in which neural functions or neurodegenerative diseases are enriched in all of the three group, although with different terms (Figures 3E–G). The group down aging genes in group iv do not enriched any functions with significance.

**FIGURE 4:**
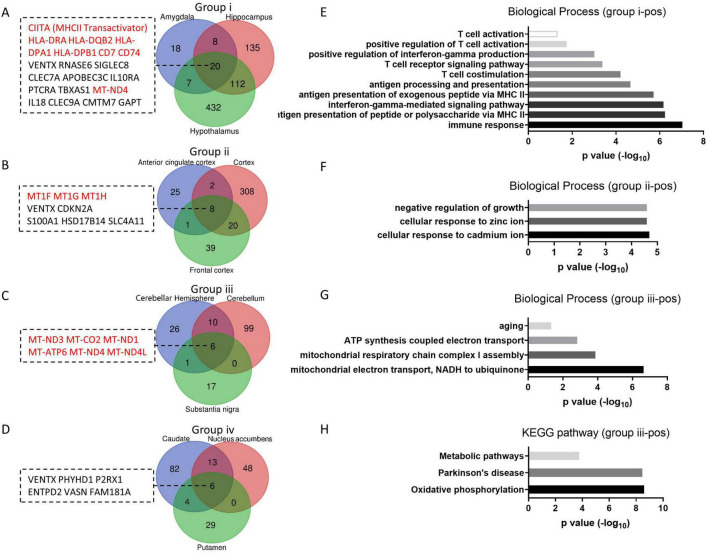
Group up aging genes and enriched functions. **(A–D)** The number of group up aging genes in each group. Venn diagrams of positive aging genes in group i to iv respectively, with group up aging genes listed. **(E–G)** Enriched biological processes of group up aging genes from group i-iii. **(H)** KEGG pathways enriched by group up aging genes from group iii.

The number of upregulated aging genes is fewer than the number of downregulated aging genes in the brain, with 20, 8, 6 and 6 aging genes in group i, ii, iii and iv respectively (Figures 4A–D). No gene is commonly upregulated across all groups. Unlike the downregulated aging genes, which regulate neuron functions as described above, the upregulated aging genes exhibit varied functions across different groups. In group i, the HLA family and CD molecules are commonly upregulated, which participate in immune activation via MCH II (Figure 4A). Functional analysis showed that all of the biological processes enriched by upregulated aging genes in group i are related to immune response terms (Figure 4E). This result aligns with previous reports indicating that the inflammatory status in the brain is heightened during aging, characterized by increased levels of immune mediators (such as MHCII, CD68, CD11, TLRs et al) (Godbout et al., 2005; Frank et al., 2006; Lucin and Wyss-Coray, 2009) and inflammatory cytokines (including IL-1β and IL-6) (Xie et al., 2003; Sierra et al., 2007; Zhang et al., 2024). In group ii, three of the eight upregulated aging genes belong to the metallothionein gene family, and the enriched functions are primarily related to ion response terms (Figures 4B–F). In addition to their role in controlling metal ion homeostasis, metallothionein proteins can function as scavengers of free radicals and provide protection against inflammation (Inoue et al., 2009; Swindell, 2011). Therefore, it is likely that metallothioneins elevate levels in the cortex and serve to prevent immune activation in group ii. In group iii, all six upregulated aging genes are chondriogenes (Figure 4C), which are important for energy production through the respiratory chain (Figure 4G), and play crucial roles in age-related disease or metabolic disorders (Figure 4H). However, the processes of mitochondrial translation are significantly downregulated in group iii during aging (Figure 3D), which may result in inefficient translation of the upregulated aging-related genes in this group. The upregulated aging genes in group iv do not belong to the three categories described above and do not enriched any functions with significance.

Altogether, the results indicate that during brain aging, the predominantly upregulated genes in each group differ, leading to distinct functional enrichment. Meanwhile, the downregulated genes are associated with neural function or neurodegenerative diseases in most tissues. In group iv, there are only a few “group down aging genes” and “group up aging genes” with no function enrichment, suggesting limited changes in the basal ganglia during aging.

### 3.3 Brain aging genes contribute to neurodegenerative diseases

Neurodegenerative diseases such as Alzheimer’s disease (AD), Parkinson’s disease (PD) and Huntington’s disease are well-known geriatric diseases associated with brain aging. It is plausible that the genes involved in brain aging also contribute to the development of these neurodegenerative diseases. To explore this possibility, we analyzed the expression of aging genes in patients with AD or PD. We selected GEO datasets from two studies(Berchtold et al., 2008; Zheng et al., 2010), each providing mRNA profiles from two different brain regions, which allowed us to analyze both tissues simultaneously and observe differences between them. The expression levels of brain aging genes were compared between AD or PD patients and the matched healthy control subjects (Supplementary Table 1). It is hypothesized that if the dysregulation of a gene contributes to both brain aging and neurodegeneration, it should exhibit synchronous changes during these two processes. Therefore, we screened for genes that simultaneously up- or downregulated in aging brain and neurodegenerative diseases.

**FIGURE 5:**
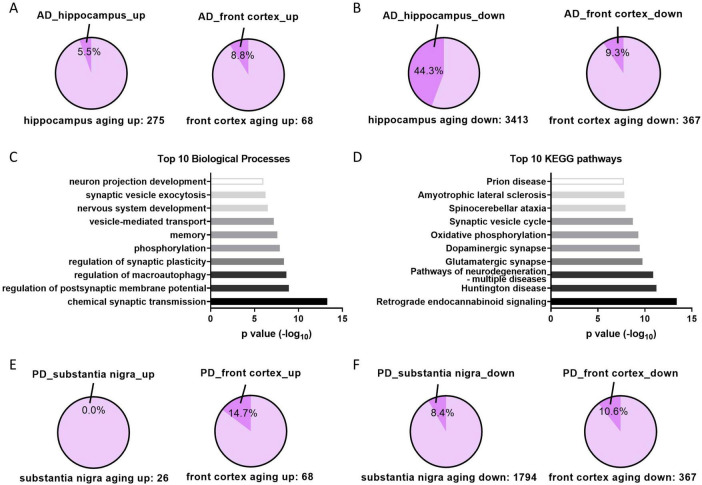
Aging genes in AD and PD patients. **(A)** The proportions of upregulated aging genes that further upregulated in corresponding tissues of AD patients. **(B)** The proportions of downregulated aging genes that further downregulated in corresponding tissues of AD patients. **(C,D)** Top 10 of enriched biological processes or KEGG pathways of aging and AD downregulated genes in hippocampus (genes in the 44.3% in panel **B**). **(E,F)** Same analysis as panels **(A,B)** in indicated tissues of PD patients.

Among the upregulated aging genes in tissues, 5.5% in the hippocampus and 8.8% in the front cortex are further upregulated in AD patients (Figure 5A), whereas 44.3 and 9.3% of downregulated aging genes are further downregulated in AD (Figure 5B), suggesting downregulated aging genes in hippocampus contributes the most to the development of AD. Functional and pathway analysis of the 44.3% syntropic downregulated genes in hippocampus displayed that these genes are enriched in neuron functions especially synaptic transmission and neurodegenerative diseases (Figures 5C, D). The same analysis was performed in PD using front cortex and substantia nigra datasets. The result showed fewer aging genes synchronously deregulated in PD patients as compared to AD, with proportions ranging between 0 and 14.7% (Figures 5E, F). However, since transcriptome of PD hippocampus is not available, it is unknown whether downregulated aging genes in hippocampus are further downregulated in PD hippocampus.

### 3.4 High physical activity retards hippocampus aging

**FIGURE 6:**
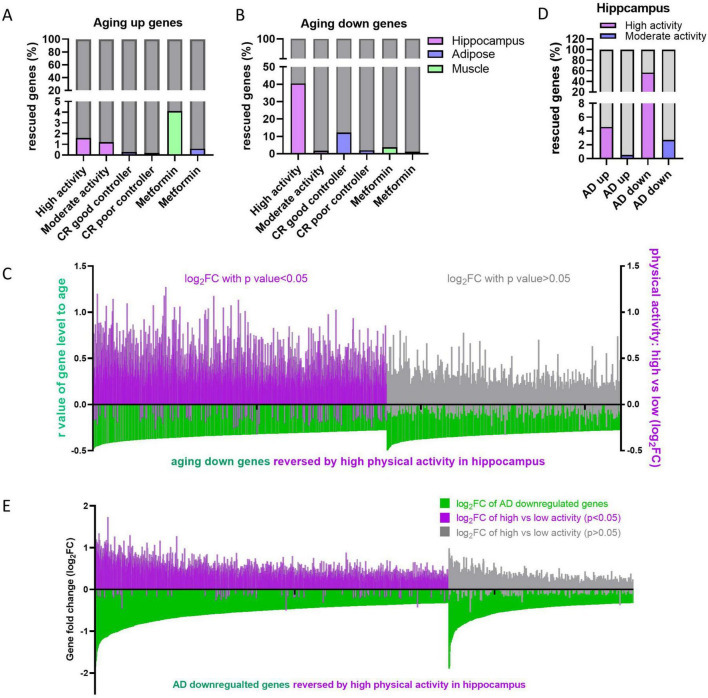
Effects of retard aging solutions on aging and AD gene expression. **(A,B)** Proportion of reversed aging up **(A)** or down **(B)** genes by indicating retard aging solutions in indicating tissues. **(C)** High physical activity rescues downregulated aging genes in hippocampus. The gene level-age *r*-values of downregulated aging genes in hippocampus were displayed in green bars. The log_2_FC of corresponding genes in high physical activity individuals versus low ones were displayed in purple (*p* < 0.05) or gray (*p* > 0.05). **(D)** Proportion of AD up- or downregulated genes reversed by high or moderate physical activity in hippocampus. **(E)** High physical activity rescues AD downregulated genes in hippocampus. In each analysis, aging genes that did not detected in the corresponding retarding aging solutions were excluded.

Researchers have been exploring solutions for healthy aging and reducing geriatric diseases for decades. Various approaches have been reported to slow down aging, including physical activity (Colcombe et al., 2004; Tsai et al., 2015), calorie restriction (CR) (Wahl et al., 2018) or intervening metabolism by using drugs (Cabreiro et al., 2013). We were curious whether these anti-aging methods could reverse the tissue-specific deregulation of aging genes. To address this question, transcriptome datasets from anti-aging studies (Bolton et al., 2017; Kulkarni et al., 2018; Berchtold et al., 2019) were obtained from the GEO database, and the expression levels of aging genes were analyzed in the physical activity group, CR group, and metformin group. Subjects in physical activity group were divided into two groups including high physical activity (actigraphy > 2.1, × 10e5 counts/day) and moderate physical activity (1 < actigraphy ≤ 2.1, × 10e5 counts/day), whereas subjects in CR group were divided into good controller (maintained weight loss) and bad controller (regained weight) (Supplementary Table 1) as previous reports (Bolton et al., 2017; Berchtold et al., 2019). The gene expression profiles of different tissues were detected in these studies, with hippocampus in physical exercise study (Berchtold et al., 2019), adipose in CR study (Bolton et al., 2017), muscle and adipose in metformin study (Kulkarni et al., 2018), hence, the aging genes in the corresponding tissues were used for rescue analysis. We found that very small proportions (less than 5%) of the upregulated aging genes were rescued (Figure 6A), whereas the rescue proportions of the downregulated aging genes varied from about 1–40%. Specifically, high physical activity rescued 40.51% of downregulated aging genes in the hippocampus, while the CR good controller group rescued 12.28% in adipose tissue (Figure 6B). In addition, most of the downregulated aging genes in hippocampus are upregulated in high physical group, although some of them with no statistical significance (*p* > 0.05) (Figure 6C). Next, we further investigated whether physical activity could reverse the deregulated genes in AD. Similarly, high physical activity reversed 56.78% of the downregulated genes in the AD hippocampus, whereas other groups showed only small proportions of genes being reversed (Figure 6D). Most of the downregulated genes in AD were upregulated in high physical activity population compared to low physical activity population, although some of them showed no statistical significance (Figure 6E). Collectively, these results suggest high physical activity is an effective solution for healthy aging and reducing geriatric diseases.

**FIGURE 7:**
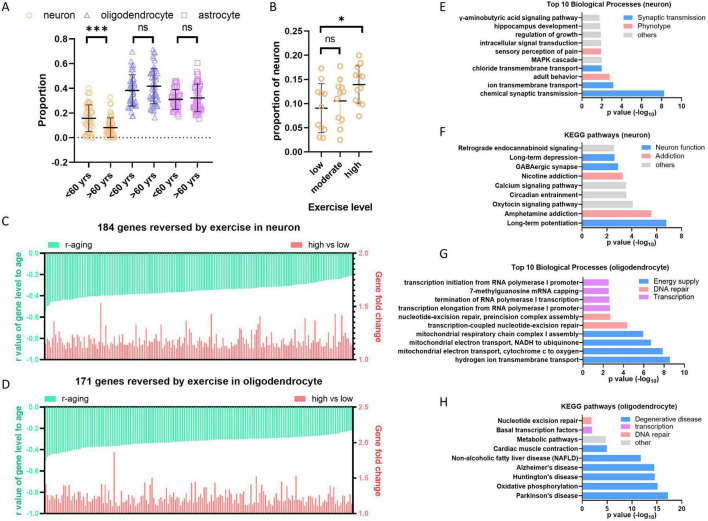
High level exercise reverses hippocampus aging. **(A)** Neuron cells decreased in hippocampus during aging. Cell dissection were performed with hippocampus bulk sequence data from GTEx using CIBERSORTx. **(B)** High level exercise increases the proportion of neuron cells. Cell dissection were performed with RNAseq data from physical activity study (GSE110298) using CIBERSORTx. **(C,D)** High level exercise reversed negative aging genes in neuron and oligodendrocyte cells of hippocampus. Cell type-specific gene expression profiles of hippocampus from GTEx and GSE110298 were generated by “high-resolution expression imputation” function of CIBERSORTx. Exercise reversed negative aging genes in neuron or oligodendrocyte were showed respectively (*p* < 0.05, fold > 1.1). **(E,F)** Enriched biological process and KEGG pathways by reversed genes in neuron. **(G,H)** Enriched biological process and KEGG pathways by reversed genes in oligodendrocyte. Student’s *t*-test, *p < 0.05; **p < 0.01; ***p < 0.001.

The central nervous system (CNS) is mainly composed of neuron, astrocyte, oligodendrocyte, vascular cell, microglia and oligodendrocyte progenitor cell (OPC). We are curious about which types of cells are affected by aging and anti-aging solutions. To address this question, we utilized a convenient digital cytometry tool called “CIBERSORTx,” which is a machine learning method that allows for the inference of cell fractions and cell-type-specific gene expression profiles without physical cell isolation (Newman et al., 2019). We generated signature matrix for CNS cells using single-cell sequencing data of brain tissue (GSE84465) (Darmanis et al., 2017), which includes main types of cells in CNS. Then, cell fractions were dissected from bulk tissue transcriptome using this signature matrix by CIBERSORTx. The hippocampus data from GTEx was analyzed to explore aging effect, whereas hippocampus data from physical activity was analyzed to explore anti-aging effect. The result showed that the proportion of neuron cells in the hippocampus decreases after age 60, while oligodendrocytes and astrocytes do not show significant changes (Figure 7A). Neuron loss is a well-known phenomenon in human neurodegenerative diseases (Andreone et al., 2020). Our results suggest that neuron loss also happen during aging without neurodegenerative disease, and this conclusion is supported by a research on chimpanzees, which observed regional neuron loss during health aging (Edler et al., 2020). In the exercise groups, older individuals with high physical activity, but not moderate physical activity, retained more neuron cells than those with low physical activity (Figure 7B), indicates that high physical activity prevent neuron loss during aging.

To further find out which kind of cells contribute most to the expression decreasing during hippocampus aging, we performed the “high-resolution expression imputation” analysis in CIBERSORTx using 3413 downregulated aging genes in hippocampus (Supplementary Table 5). Both neuron and oligodendrocyte primarily contributed to the decrease in aging gene expression, with 1601 out of 3413 genes decreased in neurons and 1908 out of 3413 genes decreased in oligodendrocytes. Among these genes, 184 out of 1601 genes are rescued in neurons from the high physical activity group, and 171 out of 1908 genes are rescued in oligodendrocytes (Figures 7C, D and Supplementary Tables 7, 8). The function analysis of these genes by DAVID (Huang da et al., 2009) displayed that genes reversed in neuron cells enrich in synaptic transmission, potentially ameliorate neuron function and addressing addiction (Figures 7E, F). Genes reversed in oligodendrocyte enrich terms of mitochondrial energy supply, DNA repair and transcription (Figure 7G). Additionally, The KEGG pathways associated with degenerative diseases are significantly enriched (Figure 7H), indicating that the rescue of genes involved in mitochondrial energy supply by high level exercise is beneficial for preventing degenerative diseases.

Altogether, the results from hippocampus study revealed that high physical activity rescues neuron loss during aging and also rescues a significant proportion of downregulated genes. Since hippocampus plays a key role in memory and learning, high physical activity may prevent the cognitive impairment and degeneration disease by diminishing decrease of important genes related to synaptic function and mitochondrial energy supply in neuron and oligodendrocyte.

## 4 Discussion

Aging is a complex physical process characterized by multiple changes in tissues and organs, including neurodegeneration, cognitive decline, increases in blood pressure, declines in other cardiovascular functions, loss of muscle strength and mass, reduction in metabolic rate and etc. (Garatachea et al., 2015). These are normal aging processes without pathological changes; however, they increase the risk of geriatric diseases. An important question is whether there are common aging genes across different tissues and organs, whose expression levels gradually increase or decrease with age, leading to the functional decline of tissues and organs. To address this question, we analyzed the transcriptome alterations of 32 tissues/organs during aging. We found an astonishing difference between brain and body tissues during aging, with most genes showing a negative association with age in the brain but not in other body tissues. It has been reported that the variations between transcriptome profiles are greater among tissues than among individuals (Melé et al., 2015), which may result in varying aging transcriptomes. Specifically, the body aging transcriptomes are diverse whereas the brain aging transcriptomes are similar (Figure 1), so we focused on the brain aging transcriptomes in this study.

### 4.1 Downregulation of neurotransmission genes during brain aging and degeneration

Large amount of genes downregulated during brain aging (Figure 1 and Supplementary Table 5). Most of these genes evolve in neuron function regulation, including synaptic transmission, action potential, mitochondrial translation, etc. (Figure 3). Among these functions, synaptic transmission and action potential are innate functions of neuron, while the others have also been reported to be important for neuron function. The mitochondrial oxidative phosphorylation system (OXPHOS) transforms energy from reducing equivalents into ATP. Thirteen proteins in the OXPHOS are encoded by the mitochondrial genome (mtDNA). Although the transcription of some mtDNA is increased in group iii (Figure 4), the downregulation of mitochondrial translation would hinder mRNA translation (Figure 3), leading to mitochondrial disorders that affect the central nervous system (Area-Gomez et al., 2019). Neurons are terminally differentiated and long-lived cells. There are many aged or damaged cellular organelles, such as mitochondria, as well as aggregated proteins that need to be turned over. Both autophagy (Stavoe and Holzbaur, 2019) and ubiquitin-proteasome pathway (Bingol and Sheng, 2011) are employed to maintain neuronal homeostasis. Hence, the gradual downregulation of these functions during aging likely drives age-related neurodegeneration and cognitive decline in healthy individuals.

Furthermore, the downregulated aging genes exhibited significant overlap with downregulated genes observed in the hippocampus of AD patients, whereas the other groups showed limited overlap (Figure 5). Given that the control group of AD or PD patients are age-matched people, the overlap genes are further up- or downregulated in addition to age-related alteration. Therefore, it appears that the downregulated aging genes in hippocampus play a significant role in AD development, whereas the other sets of aging genes contribute to AD or PD with limited effects.

### 4.2 High physical activity and effective calorie restriction partially reverse aging transcriptome

Several methods have been reported as effective solutions for anti-aging, including calorie restriction (Most et al., 2017), physical activity (Garatachea et al., 2015) and metformin treatment (Soukas et al., 2019). All of the mentioned retard aging solutions have been shown to improve deteriorated physical function in humans. Calorie restriction extends lifespan in model organisms and reduces age-associated parameters, including fat mass, blood pressure, inflammation and insulin levels, which are risk factors for diabetes, cardiovascular disease or cancer (Wei et al., 2017). Physical exercise improves cardiorespiratory fitness, muscle function, neurodegeneration and etc. (Garatachea et al., 2015). Metformin, originally prescribed as an oral hypoglycemic medication for type 2 diabetes, has been later found to mimic the effects of calorie restriction, leading to an extension of lifespan in model organisms and a retardation of aging and aging-related diseases in humans (Soukas et al., 2019). As described above, the expression levels of many genes changed during aging. We are curious whether the three retard aging solutions could reverse these aging-related gene changes. According to our results, only high physical activity and good controllers during calorie restriction, but not moderate physical activity, bad controllers during calorie restriction, or metformin therapy, could reverse the downregulated aging genes or the downregulated genes in AD (Figure 6). High physical activity also prevents neuron loss during aging (Figure 7). It is unexpected that metformin, as a calorie restriction mimic, did not reverse the downregulated aging gene as effectively as calorie restriction did. One possible reason is that individuals taking metformin only experienced a modest weight loss of 1.5% (Kulkarni et al., 2018), whereas good controller in calorie restriction lost 13% (Bolton et al., 2017). Therefore, subjects in metformin treatment group are more similar to poor controller group. It gives us a clue that when retard aging using physical activity or calorie restriction, people need to exercise at least > 2.1 × 10e5 counts/day measuring with actigraphy (equivalent to 42 min/day moderate-high activity), or keep certain amount of weight loss (about 13%) for overweight individuals during calorie restriction (Bolton et al., 2017; Berchtold et al., 2019). In addition, the upregulated aging genes were not reversed by any retard aging solutions, suggesting re-expression of the downregulated genes is more effective for anti-aging than suppress genes upregulated during aging.

### 4.3 Limitation of this study

In this study, although we utilized different datasets to validate the aging genes and enhance the authenticity of our findings, a significant limitation is the lack of functional validation experiments. This challenge is common in aging research, as obtaining clinical samples, particularly from the human brain, is difficult. While animal models, such as aging mice, could serve as an alternative, they are also time-consuming. In our study, we analyzed data exclusively from humans and do not know whether these aging genes are deregulated in animal models. We attempted to validate our findings using stress-induced senescent cells. We confirmed that two aging genes regulate cellular aging (Figure 2), suggesting that at least some of the top genes identified can influence aging and warrant further investigation in the future.

## 5 Conclusion

In conclusion, there is no common aging genes between brain and the other parts of the body since their transcriptome alterations vary widely. The main factor for brain aging is the downregulation of many genes, which may also lead to neurodegeneration. High physical activity effectively reversed the downregulated aging genes, making it an attractive anti-aging solution.

## Data Availability

The original contributions presented in this study are included in this article/Supplementary material, further inquiries can be directed to the corresponding authors.

## Footnotes

1
https://www.gtexportal.org/home/datasets
